# A Comparative Study between Supraorbital Keyhole and Pterional Approaches on Anterior Circulation Aneurysms

**DOI:** 10.21315/mjms2018.25.5.6

**Published:** 2018-10-30

**Authors:** Pravinna Genesan, Mohammad Saffari Mohammad Haspani, Saiful Razman Mohd Noor

**Affiliations:** 1 Department of Neurosciences, School of Medical Sciences, Universiti Sains Malaysia, 16150 Kubang Kerian, Kelantan, Malaysia; 2 Department of Neurosurgery, Hospital Kuala Lumpur, Jalan Pahang, 50586 Kuala Lumpur, Malaysia; 3 Neurology & Neurosurgery Centre, Prince Court Medical Centre, 50450 Kuala Lumpur, Malaysia; 4 Thomson Hospital Kota Damansara, 47810 Petaling Jaya, Selangor, Malaysia

**Keywords:** supraorbital, craniotomy, aneurysm, neurosurgery, clipping

## Abstract

**Background:**

Many different conventional approaches such as the frontal and pterional approaches are used to access anterior circulation aneurysms. Recently, the supraorbital approach has been widely applied to the treatment of anterior circulation aneurysms. This study was done to evaluate which approach (pterional or supraorbital) has better outcomes in terms of residual neck post-clipping, cosmetic satisfaction, scar tenderness, complications and functional outcomes.

**Methodology:**

A total of 123 patients were recruited into this study, comprising 82 patients who underwent a pterional approach and 41 patients who underwent a supraorbital approach. Computed tomography angiograms, the modified Rankin Scale, and the visual analogue scale were administered at 6 months to look for residual aneurysm, functional outcomes, scar tenderness, and cosmetic satisfaction. Complication data were collected from patients’ case notes.

**Results:**

The mean operating time for the pterional group was 226 min, compared to supraorbital group, which was 192 min (*P* = 0.07). Cosmetic satisfaction was significantly higher (*P* = 0.001) in the supraorbital group. There was no significant difference between the supraorbital and pterional groups’ scar tenderness (*P* = 0.719), residual aneurysm (*P* = 0.719), or functional outcomes (*P* = 0.137), and there was no significant difference between the groups in terms of intra-operative and post-operative complications.

**Conclusions:**

The supraorbital group had better cosmetic outcomes and shorter operating times compared to the pterional group.

## Introduction

**Figure 1:**
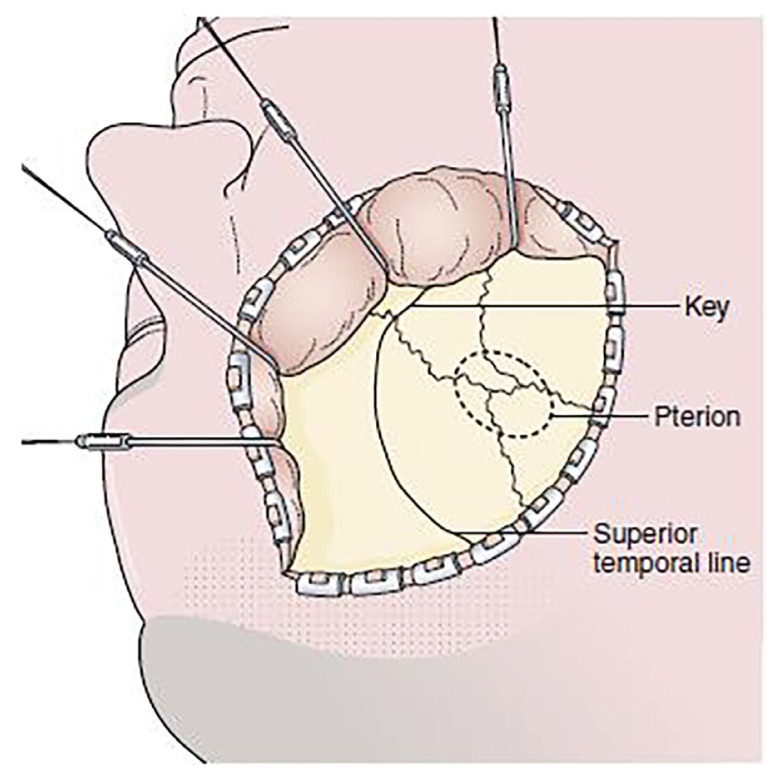
Pterional approach

**Figure 2:**
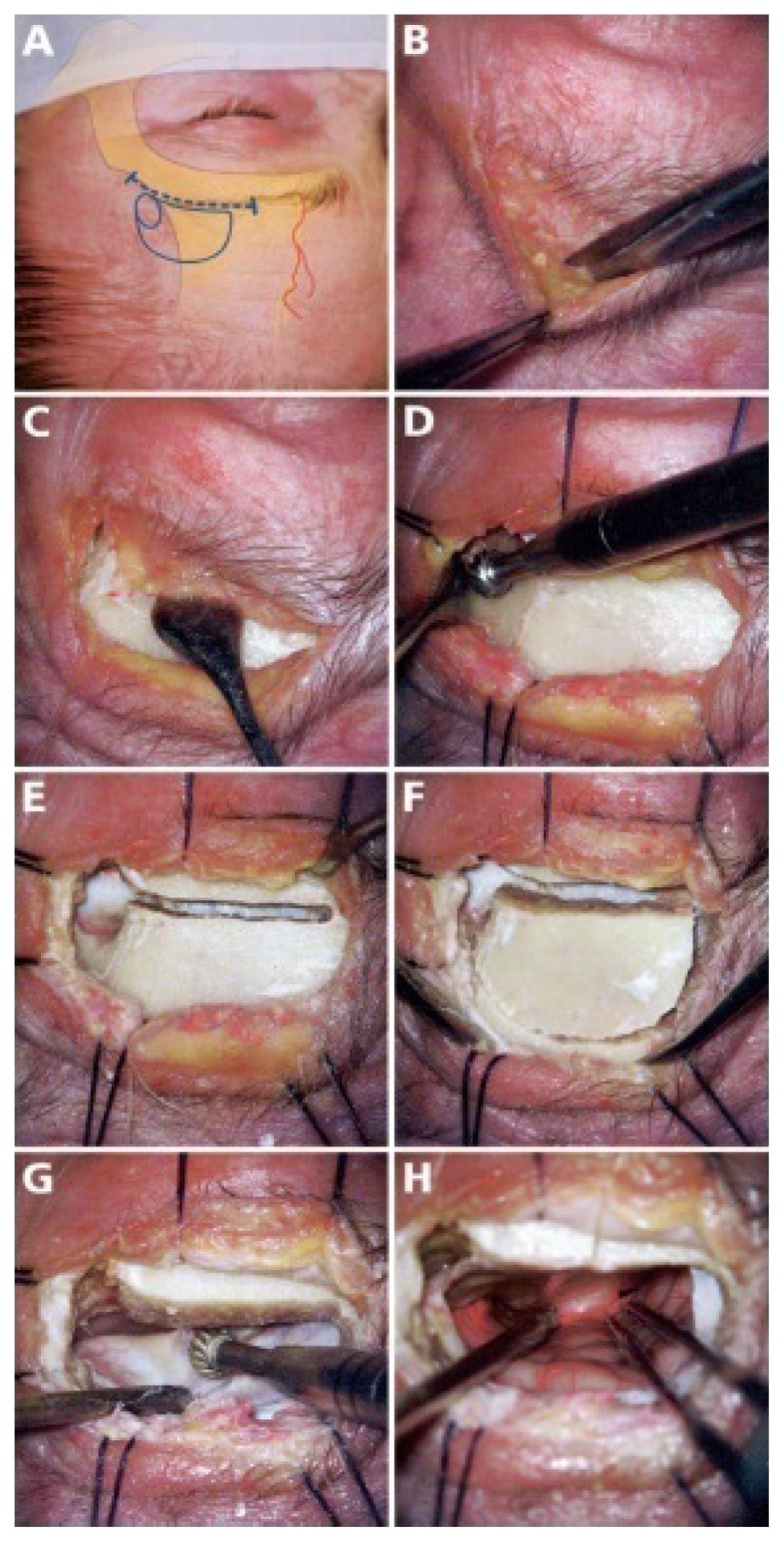
Supraorbital approach

Many different conventional approaches such as the frontal and pterional approaches are used to access anterior circulation aneurysms. These approaches provide excellent exposure to the anterior and middle cranial fossa (1). The major limitation of these approaches is that an extended opening is needed to expose the orbital rim. In recent decades, the development of surgical instruments and microsurgical skills has allowed neurosurgeons to use smaller and more specific approaches to treat these lesions in the same region (1–2). One of these new approaches, supraorbital subfrontal craniotomy, has been reported to offer a minimally invasive approach to a variety of lesions. Recently, this approach has been widely applied to the treatment of anterior circulation aneurysms (Figure 1, Figure 2).

## Methodology

This is a single-institution, non-randomised, cross-sectional study. The data were collected retrospectively from patients admitted to the elective or emergency list for aneurysm clipping. The study was conducted between 1 January, 2011 and 1 May, 2016, and the operating physicians were Datuk Mohammad Saffari and Saiful Razman. A total of 123 patients were recruited into this study. The data were collected from the patients’ medical records, patient interviews, and by performing computed tomography (CT) angiograms on the patients; the size of the residual aneurysm was also calculated with CT angiograms. Scar tenderness and cosmetic satisfaction were evaluated at 6 months post-surgery using the visual analogue scale (VAS), scored between 1 and 5 (scar tenderness: 1 = no pain, 5 = severe pain; cosmetic satisfaction: 1 = very satisfied, 5 = not satisfied).

## Results

Out of the 123 patients, 41 patients underwent the supraorbital approach for anterior circulation aneurysm clipping while 82 patients underwent the pterional approach. Fifty six patients were male and 67 were female. The majority of the patients were female (54.5%), and the mean age of all patients was 54.95 (12.71) years old. The youngest patient was 26 years old and the oldest patient was 82. The peak age in our study was 40 to 60 years old. Among the 123 patients, 67 (54.47%) had hypertension, 68 (55.30%) had comorbidity, 52 (42.28%) were classified as the World Federation of Neurological Surgeons (WFNS) 1, and 56 (45.53%) were classified as Fisher Grade 3. The most common aneurysm type was the internal carotid artery (ICA) aneurysm. All of the aneurysms were clipped successfully.

The patients who underwent supraorbital clipping appeared to have better modified Rankin Scores (mRSs) at the end of 6 months compared to those who underwent pterional clipping. However, when Pearson’s chi-square test was applied to analyse the association between the treatment group and mRS outcomes, there were no significant differences between the treatment groups (*P* = 0.137). When Pearson’s chi-square test was applied to analyse the difference between the treatment groups and the mRS outcomes from the selected good WFNS group, it was noted that there was no significant difference between the treatment (supraorbital and pterional) groups and their mRS outcomes (*P* = 0.571). Similar results were reproduced in the group with poor WFNS presentation (*P* = 0.248). Several complications were noted post-operatively, but there was no significant difference when both groups were compared. The complications included frontal muscle weakness (*P* > 0.995), frontal numbness (*P* = 0.614), hyposmia (*P* > 0.995), meningitis (*P* = 0.860), and rhinorrhoea (*P* = 0.156).

The operating time was markedly reduced in the supraorbital group, with a *P*-value of 0.007. There was no significant difference between the supraorbital and pterional groups in terms of scar tenderness (*P* = 0.719) and residual aneurysm (*P* = 0.719). Cosmetic satisfaction was significantly higher in the supraorbital group, with a *P*-value of 0.001. In our study, the patients in the under-40-age group had good mRS outcomes, with 14/18 patients (77.8%). On the other hand, only 65/105 (64.8%) of the patients over 40 had better mRS outcomes at 6 months. However, when Pearson’s chi-square test was applied, we could not find any significant statistical differences in the outcomes (*P* = 0.279).

Patients with anterior cerebral artery (ACA) aneurysms who underwent supraorbital clipping had better mRS outcomes at the end of 6 months compared to the pterional clipping group (*P* < 0.001), but 77% of the supraorbital patients with ACA aneurysms had good pre-clipping WFNS scores (0–2), and only 34.62% of the patients in the pterional group had good WFNS scores (0–2). There was also a statistically significant difference between good WFNS grades and good mRS outcomes at 6 months (*P* < 0.001) as well as a statistically significant difference between good Fisher grades and good mRS outcomes at 6 months (*P* < 0.002). Nonetheless, meningitis and requiring an external ventricular drain (EVD) were associated with poor outcomes (*P* < 0.001).

## Discussion

Frontotemporal craniotomy with a pterional approach was first described by Yasargil and Fox (3). Since then, this technique has replaced bifrontal and frontolateral craniotomies for access to anterior circulation aneurysms. The standard pterional approach requires a wide exposure of the brain, which can cause an increase in surgical morbidity not related to the lesion. Some patients who undergo the pterional approach complain of post-operative cosmetic impairments in the frontotemporal area resulting from the large skin incision scar, depression of the bone flap, inappropriate repair of burr holes, or temporal muscle atrophy.

Recently, applying the concept of minimal invasiveness to neurosurgical procedures has gained acceptance among neurosurgeons, with the understanding that keyhole surgery is not the miniaturisation of any standard technique but rather the natural evolution into a more precised and refined procedure. Various modified and combined pterional approaches to skull-base lesions have been reported (4–8).

Cohen et al. (4) advocated the keyhole concept in neurosurgery and have used supraorbital mini craniotomy extensively to treat a variety of lesions. Eyebrow skin incisions and supraorbital keyhole craniotomy were originally recommended for microsurgery on various cerebral aneurysms and tumours located in the supra- and parasellar regions (5). One of the most important goals of the supraorbital keyhole approach is the minimisation of brain exposure via limited and more specific craniotomies. Exposing the brain tissue for several hours during extended craniotomies always leads to injury of the brain’s surface from the non-physiological surroundings, such as the air in the room, the irrigation media, and the cover material. The supraorbital keyhole approach offers minimal brain exposure to air and accidental surgical trauma. Moreover, brain retraction is minimised or absent in this procedure. This significantly decreases approach-related surgical morbidity and shortens hospitalisation. In terms of operation time, experienced surgeons need less time to perform the surgery using pterional craniotomy. However, manipulation of the temporal muscle and performing a relatively larger size of craniotomy may require more time.

The anatomy of the suprasellar area offers several advantages for keyhole approaches from the anterior. The suprasellar area is bordered by the mesencephalon posteriorly and by the temporal lobes laterally. Therefore, approaches from these sides require retracting brain tissue. Anteriorly, however, there are several windows into the suprasellar area that do not require brain retraction. Supraorbital craniotomy offers access to the circle of Willis, and dissection of the Sylvian fissure can be performed easily. The burr hole in the frontal bone may be expanded towards the back or downwards if additional exposure of the middle fossa is desired. In addition, orbital rim osteotomy provides a direct view of the anterior cranial fossa, which includes the sellar and suprasellar regions, with even less brain retraction (6). If the frontal process of the zygomatic bone is removed, the structures of the middle fossa can be well appreciated. This approach allows for the clipping of aneurysms located in the ipsilateral internal carotid artery, the anterior communicating artery, the middle cerebral artery, and even in the contralateral internal carotid artery. According to (7), there were no approach-related complications in 139 supraorbital keyhole craniotomies performed for anterior circulation aneurysms.

At Kuala Lumpur Hospital, we performed surgery on 123 cases of intracranial anterior circulation aneurysm. The patients underwent microsurgical clipping between 1 January, 2011 and 1 May, 2016. This study is comparable to a previous study (2), which had 113 patients. In that study, 41 patients underwent supraorbital clipping of the aneurysms, and 82 patients underwent pterional clipping. Pterional clipping was chosen more in that study because the pterional approach was used in more cases in Kuala Lumpur Hospital in the past; the supraorbital technique has only been performed in the past few years. The mean age in that study was 54.95 years old, and a majority of the patients (35%) were 50 to 59 years old, followed by 20% aged 60 to 69. The smallest age group (3%) was patients aged 20 to 29 years old. The youngest patient was 26 and the oldest was 82 years old. The peak age in our study is 40 to 60 years old, which is comparable with most published data (8).

More than half of the study population was female (54.5%); usually females are more affected than males (9).

There were 41 males and 41 females in the pterional group, and 15 males and 26 females in the supraorbital group. More female patients underwent supraorbital clipping compared to male patients, which may be due to cosmetic reasons. In Malaysia, there is no available data for intracranial aneurysms, but in this study, Malays (87%) were more frequently affected, followed by Chinese and Indian, which may, in fact, reflect the diversity of the local population rather than any racial predominance in aneurysm ruptures.

In this study, 55.3% of the patients had co-morbidity. Among the 123 patients, 54.5% of them had hypertension, 4.8% had diabetes, and 2% had ischemic heart disease.

Hypertension can be considered an important risk factor for subarachnoid hemorrhage (SAH) and possibly for aneurysm formation and fatal aneurysm rupture (10), which correlates with our findings that 54.5% of our patients had hypertension. Hypertension is a risk factor for de novo aneurysm formation (10). Diabetes mellitus is associated with substantial reduction of the risk of SAH (10), and in our study, only 4.8% of the patients had diabetes. There is no previous study that shows a correlation between patients with ischemic heart disease and subarachnoid haemorrhage, and in our study, only 2% of the patients had ischemic heart disease.

In aneurysmal SAH, there are three variables that are most closely related to outcomes: the neurological condition of the patient on admission, age, and the amount of extravagated blood seen on CT scans. The neurological condition, particularly the level of consciousness, is the most important determinant of outcomes after SAH. Therefore, since the neurological condition can change during the clinical course after SAH, it is important to have a reliable (i.e., high inter- and intra-observer agreement) and valid (i.e., good relationship to outcomes) grading system for unequivocal and understandable documentation. Since grading scales based on the Glasgow Coma Scale (GCS) have the advantage of reasonable inter-observer agreement, a committee of the WFNS proposed a grading scale of five levels, essentially based on the GCS, with focal deficits making up one extra level for patients with a GCS of 14 or 13. The cut-off points in the WFNS scale are based on consensus, not on formal analysis (11). WFNS 1 are patients with full GCS, WFNS 2 are patients with GCS 13–14, WFNS grade 3 are patients with GCS 13–14 with major focal deficits, WFNS 4 are patients with GCS 7–12, with or without major focal deficits, and WFNS 5 are patients with GCS 3–6, with or without major focal deficits. Most of the patients in our study fell under grade 1 (42.28%) on the WFNS grading scale, followed by grade 5 (17.07%), grade 2 (15.45%), grade 4 (13.82%), and the lowest numbers were patients with grade 3 (8.94%) and grade 0 (2.44%). Patients with WFNS grade 1 had a 70% survival rate, grade 2 had a 60% survival rate, grade 3 had a 50% survival rate, grade 4 had a 40% survival rate, and grade 5 had a 10% survival rate. In this study, most of the patients had good WFNS scores, which means they had a good survival rate. In our population, 54.9% had a good score (WFNS 0–2), but only 70% had a good WFNS score in the supraorbital group; there was no significant difference between the pterional and supraorbital groups with good scores (WFNS 0–2) and poor scores (WFNS 3–5) (*P* = 0.09).

All of the patients in the study were also graded on the amount of blood in the CT scan. Fisher grading was used for this study. Fisher grade 1 is when no subarachnoid blood is detected, Fisher grade 2 is assigned when there is a diffuse or vertical layer of subarachnoid blood less than 1 mm thick, Fisher grade 3 is when there is a localised clot or a vertical layer less than or equal to 1 mm, and Fisher grade 4 is when there is an intracerebral or intraventricular clot with a diffuse subarachnoid haemorrhage (12). Fisher grade 3 has higher chances of vasospasm, followed by grade 4, and then grade 2 and grade 1. In this study, 46% of patients fell under Fisher grade 3, 33% fell into grade 4, 16% fell into grade 1, and 4.8% fell into grade 2. In this study, most of the patients were classified as Fisher grade 3, where there is high risk of vasospasm. There was no statistically significant difference in the Fisher scores of the pterional group and the supraorbital group (*P* = 0.428).

Most patients’ intracranial aneurysms were located at the ICA (44.72%), followed by the ACA (35.77%) and the middle cerebral artery (MCA) (19.51%). There was an almost equal percentage of cases in the supraorbital and pterional groups in the ACA and ICA locations, but there was only 7.30% of MCA cases in the supraorbital group; 25.61% of patients in the pterional group underwent pterional clipping. This is because the MCA artery is considered unsuitable for the supraorbital technique; if there is a MCA bifurcation aneurysm, the M1 segment is too long, and the direction of the dome in a MCA aneurysm is lateral or caudal to surgical view.

### Correlations between Complications, Residual Aneurysms, and Post-operative Functional Outcomes in the Treatment Groups

This study showed that the supraorbital group had better cosmetic satisfaction compared to the pterional group. Cosmetic satisfaction was evaluated using the patients’ subjective response, with scores between 1 and 5. The descriptions were rated on the VAS, with “none” and “most severe imaginable” (cosmetic satisfaction; 1 = very satisfied, 5 = not satisfied). In the supraorbital group, all of the patients chose VAS 1, but in the pterional group, 76.8% had VAS 1, 19.5% had VAS 2 and 3.7% had VAS 3.

Statistically, there was a significant difference between the pterional and supraorbital groups when cosmetic satisfaction was compared (*P* < 0.001). The most common cosmetic problem after pterional craniotomy was the presence of depressed deformities in the frontotemporal area that result from temporal muscle atrophy. Conventional large frontotemporal craniotomy uses multiple burr holes and needs rongeuring of the temporal squama. Depression of the bone flap or atrophy of the temporalis muscle results in a skin dent in the forehead. Patients sometimes complain of a limited ability to open the mouth and pain during chewing that results from temporal muscle resection and contracture after a conventional frontotemporal craniotomy. Small craniotomy, which is fixed with a plate and screw, is unlikely to cause cosmetic problems. Preservation of the superficial temporal artery, the frontal branches of the facial nerve, and the supraorbital nerve and artery minimises the risk of wound healing problems. A short skin incision is later hidden by the eyebrow. Therefore, the post-operative cosmetic results observed in this approach were quite acceptable and, in most patients, were excellent (2).

There were no statistically significant results when scar tenderness was compared between the supraorbital and pterional groups (*P* = 0.719), as shown in Table 15. The VAS is a simple and frequently used method for the assessment of variations in intensity of pain used in this study. Scar tenderness and cosmetic satisfaction were evaluated using the patients’ subjective response, given as a score between 1 and 5. The descriptions were rated on the VAS, using “none” and “most severe imaginable” (scar tenderness: 1 = no pain, 5 = severe pain; cosmetic satisfaction 1 = very satisfied, 5 = not satisfied). The reason why there was no difference between the groups was because patients’ scar tenderness was evaluated 6 months post-surgery, when healing had taken place.

Post-operative computed tomographic angiography (CTA) was done to look for residual aneurysms. Villablanca et al. (13) reported a CTA sensitivity of 98%–100%, compared with 95% with intra-arterial digital subtraction angiography (IADSA), suggesting a central role for CTA in the evaluation of all patients with symptomatic and potentially asymptomatic intra-arterial aneurysms. There was no statistically significant difference in residual aneurysms between the two groups (*P* = 0.719). The reason there was no difference between the two approaches was because although the skin incision, craniotomy size, and dura openings are different, both of the approaches use the standard microsurgical procedure to clip the aneurysm (14).

The mRS was used to assess patients’ functional status at 6 months post-operation (0 = no symptoms at all, 1 = no significant disability despite symptoms, able to carry out all usual duties and activities; 2 = slight disability, unable to carry out all previous activities but able to look after own affairs without assistance; 3 = moderate disability, requiring some help but able to walk without assistance; 4 = moderately severe disability, unable to walk without assistance and unable to attend to own bodily needs without assistance; 5 = severe disability, bedridden, incontinent, and requiring constant nursing care and attention). In this study, we classified the patients as having good mRS (0–2) and poor mRS (3–5). The overall outcomes in both groups were good. There was no statistically significant difference in the mRS between the two groups (*P* = 0.137), similar to a previous study by Park and Park (2). The reason there was no difference between the two approaches is because although the skin incision, craniotomy size, and dura openings are different, both of the approaches use the standard microsurgical procedure to clip the aneurysm (14), as mentioned earlier.

The mean operation time for the pterional approach was 226 min, and the mean operation time for the supraorbital approach was 192 min. There was a statistically significant difference in operation times between the two groups (*P* = 0.007), similar to a previous study (2). These operation times are because of smaller skin incisions, smaller craniotomies, and less brain exposure.

The mean blood loss for the pterional approach was 437 mL, and the mean blood loss for the supraorbital approach was 433 mL. There was no statistically significant difference in the amount of blood loss between the two groups (*P* = 0.972). This was because there is not much difference in blood loss from the skin incisions, craniotomy, and dura opening although the skin incision and craniotomy size are larger in the pterional approach. The most blood loss usually occurs during the rupture of the aneurysm.

We recorded post-operative complications at 6 months after surgery such as muscle weakness, frontal numbness, hyposmia, wound infection, meningitis, cerebrospinal fluid (CSF) leak from the wound, rhinorrhoea, and post-operative infarct. There was no statistically significant difference in muscle weakness between the two groups (*P* = 0.007). In the supraorbital craniotomy, the frontalis muscle is affected and in the pterional approach, the temporalis muscle is affected (2).

There was no statistically significant difference in frontal numbness between the two groups (*P* = 0.614). There was only one patient in each group that developed frontal numbness. During the skin incision using the supraorbital approach, the incision remains superficial to avoid injury to the supraorbital nerve (14).

There was no statistically significant difference in hyposmia between the two groups (*P* > 0.995). There was only one patient affected in the supraorbital group and two patients affected in the pterional group. This was because there is a chance of similar injuries between both groups during retraction and the standard microsurgical procedure to clip the aneurysm.

There was no wound infection in either group because prophylactic antibiotics were given pre-operatively, three doses were given post-operatively, and standard sterile precautions were taken in all surgeries.

There was 14% of supraorbital patients and 16% of pterional patients that developed meningitis. This was slightly higher compared to a previous study, which showed patients had only a 9.5% risk of developing meningitis (15). This is may be due to the prolonged usage of EVD and long surgeries, but there was no statistically significant difference between the two groups.

There was no statistically significant difference in intensive care unit (ICU) stay between the two groups (*P* = 0.094). This was because the standard microsurgical procedure to clip the aneurysm was applied in both the techniques, as mentioned earlier.

### Significant Findings

The supraorbital keyhole approach shows fewer cosmetic disturbances (*P* < 0.001) and shorter operation times (*P* = 0.007). The current study showed that less cosmetic disturbance. The length of the surgical procedure and hospitalisation were significantly reduced compared with standard techniques (16). The supraorbital keyhole approach offers equal surgical possibilities with less cosmetic deformity, shorter ICU stays, and shorter operation times. However, the supraorbital keyhole approach cannot be adopted as a standard approach. In our study, the patients with a mass effect on the CT scan were regarded as unsuitable for a supraorbital craniotomy. As described by (16), this approach has some limitations. In some cases of MCA bifurcation aneurysm, the M1 segment is too long, and the direction of the dome is lateral or caudal to the surgical view, which makes this approach unsuitable (2).

This type of case requires an extensive amount of dissection, and the surgical view and working space are limited by a very deep plane. In addition, small craniotomy offers a diminished opportunity for a change of plan if unexpected findings occur during surgery. This drawback can be overcome by the use of special keyhole-adapted micro-instruments and a neuroendoscope (17). However, pre-operative diagnostic imaging is of paramount importance in this procedure. Each individual patient should be evaluated for the possibility of proximal control, direction of the aneurysm, and application of the aneurysm clip. Although a transient loss of supraorbital sensation and paresis of the eyebrow can occur, this deficit appears to diminish during post-operative healing. Fortunately, in this study, there was no patient who suffered from paresis of the eyebrow.

## Conclusion

This retrospective analysis demonstrated that regardless of treatment option, functional outcomes after SAH are dependent upon clinical presentation prior to intervention. In our institution, this is determined by a WFNS grade. A good WFNS grade is a significant predictor of a good mRS outcome. Treatment methods had no significant correlation with functional outcomes after 6 months. In this study, it was shown that there was no statistically significant difference in the mRS outcomes between the supraorbital and pterional groups. Nevertheless, the treatment options for each patient should be individualised based on the clinical presentation, location and available expertise.

Regardless of the treatment used, patients with poor WFNS scores will have poor mRS outcomes at the end of 6 months. The supraorbital keyhole approach showed shorter ICU stays and fewer cosmetic disturbances. Patients presenting with Fisher grades of 1 or 2 had better outcomes compared to those with Fisher grades of 3 and 4. Absence of EVD requirement is a significant predictor of good mRS outcomes.

There is a need for a standardised prospective study of specific aneurysm types and outcomes. A detailed analysis of each aneurysm, its morphology, location and treatment options should be included. An ongoing analysis can take account of improved technological developments of treatment modalities.
